# Knockdown of Midgut Genes by dsRNA-Transgenic Plant-Mediated RNA Interference in the Hemipteran Insect *Nilaparvata lugens*

**DOI:** 10.1371/journal.pone.0020504

**Published:** 2011-05-31

**Authors:** Wenjun Zha, Xinxin Peng, Rongzhi Chen, Bo Du, Lili Zhu, Guangcun He

**Affiliations:** State Key Laboratory of Hybrid Rice, College of Life Sciences, Wuhan University, Wuhan, People's Republic of China; New Mexico State University, United States of America

## Abstract

**Background:**

RNA interference (RNAi) is a powerful technique for functional genomics research in insects. Transgenic plants producing double-stranded RNA (dsRNA) directed against insect genes have been reported for lepidopteran and coleopteran insects, showing potential for field-level control of insect pests, but this has not been reported for other insect orders.

**Methodology/Principal Findings:**

The Hemipteran insect brown planthopper (*Nilaparvata lugens* Stål) is a typical phloem sap feeder specific to rice (*Oryza sativa* L.). To analyze the potential of exploiting RNAi-mediated effects in this insect, we identified genes (*Nlsid-1* and *Nlaub*) encoding proteins that might be involved in the RNAi pathway in *N. lugens*. Both genes are expressed ubiquitously in nymphs and adult insects. Three genes (the hexose transporter gene *NlHT1*, the carboxypeptidase gene *Nlcar* and the trypsin-like serine protease gene *Nltry*) that are highly expressed in the *N. lugens* midgut were isolated and used to develop dsRNA constructs for transforming rice. RNA blot analysis showed that the dsRNAs were transcribed and some of them were processed to siRNAs in the transgenic lines. When nymphs were fed on rice plants expressing dsRNA, levels of transcripts of the targeted genes in the midgut were reduced; however, lethal phenotypic effects after dsRNA feeding were not observed.

**Conclusions:**

Our study shows that genes for the RNAi pathway (*Nlsid-1* and *Nlaub*) are present in *N. lugens*. When insects were fed on rice plant materials expressing dsRNAs, RNA interference was triggered and the target genes transcript levels were suppressed. The gene knockdown technique described here may prove to be a valuable tool for further investigations in *N. lugens*. The results demonstrate the potential of dsRNA-mediated RNAi for field-level control of planthoppers, but appropriate target genes must be selected when designing the dsRNA-transgenic plants.

## Introduction

RNA interference (RNAi), first characterized in *Caenorhabditis elegans* [1], has been developed as an effective gene-silencing tool in a wide variety of organisms [2], and double-stranded RNA (dsRNA) mediated RNAi has emerged as one of the most powerful strategies for the rapid analysis of gene function, particularly in organisms for which stable transgenesis is not available, such as insects [3]. dsRNA-mediated gene-silencing is a conserved mechanism in many eukaryotes [4], in which Dicer RNase III type enzymes bind and digest cytoplasmic dsRNAs into small interfering RNAs (siRNAs), duplexes composed of approximately 21 to 23 dsRNA nucleotides. These small RNA cleavage products then function as sequence-specific interfering RNA in transcript turnover, cleavage, and translational control [5]. Gene knockdown via dsRNA has been successfully demonstrated in several insect orders, including Diptera [6], Coleoptera [7], Hymenoptera [8], Orthoptera [9], Blattodea [10], Lepidoptera [11], [12] and Isoptera [13], and has been regularly applied in entomology to investigate RNAi mechanisms [14], and the function [15], expression and regulation [16] of gene cascades. RNAi might therefore serve as a new technique for the control of insect pests in agriculture. Nevertheless, the majority of these experiments have been carried out through dsRNA injection directly into the organisms, which is not practicable for insect pest control in the field.

For RNAi of a target gene by dsRNA to be used as an effective means of insect control, the targeted insects will have to take up dsRNA from the environment. The body of an insect is covered by a chitin exoskeleton, while the midgut of most insects is lined by the peritrophic membrane (PM), or, in Hemipterans [17], the perimicrovillar membrane (PMM). Hence, the midgut is the only portion of an insect's body that has an active interface with the physical environment. The cells of the midgut, which are responsible for nutrient absorption from the gut lumen, can take up dsRNA, and are the route through which RNAi effects would be achieved in insects [18]. In animals, the best-studied uptake mechanism of dsRNA is that of *C. elegans*. Studies with systemic RNAi defective mutants (*sid*) have resulted in the description of two proteins involved in non-cell autonomous RNAi. SID-1 is a multispan transmembrane protein that is expressed on the cell surface, thereby mediating a systemic RNAi effect [19]. It probably functions as a multimer, in cooperation and/or coordination with SID-2, transporting dsRNA passively into the *C. elegans* cells. Homologs of *sid-1-*like genes have been identified in some insect species such as *Tribolium castaneum*, *Bombyx mori* and *Apis mellifera* [3], and recently in aphids [20]. However, in the best characterized model insect, *Drosophila melanogaster*, no *sid-1-*like gene has been found.

Target regulation by siRNAs is mediated by the RNA-induced silencing complex (RISC). RISC-associated Argonaute (Ago) proteins play an essential role in mediating distinct assembly and cleavage steps of the RNA interference catalytic cycle. The Ago protein, as the principal factor in the RISC exhibiting RNA ‘slicer’ activity, is a key player in the RNAi mechanism, effecting transcriptional repression and post-transcriptional control in animals [21]. The Argonaute protein family can be divided into two classes: the Ago subfamily and the Piwi subfamily. Members of both subfamilies are defined by the presence of four domains: the N terminal domain of variable length, the central PAZ (Piwi-Argonaute-Zwille) domain that is important for binding single-stranded small RNA 3′ ends without sequence specificity, the mid domain that participates in 5′ end binding to small RNAs and binding to the 7-methylguanine (m7G) cap of target mRNAs, and the RNase H-like C-terminal Piwi domain [22]. The Piwi domain of some Ago proteins performs a crucial role in RNA slicer activity that is responsible for mRNA degradation in siRNA- and miRNA-induced gene-silencing pathways [23]. Ago proteins have been identified in several insects, including *D. melanogaster* [24] and *A. mellifer*a [25].

Systemic RNAi in plants is based on the RNA-dependent RNA polymerase (RdRp) [26] and the spread of siRNAs through the plasmodesmata [27]. RdRp orthologs are present in nematodes, but probably not in insects [28]. The absence of dsRNA amplification and RdRP in insects suggests that gene knockdown effects exhibited by feeding dsRNA to insects would be temporary. Thus, RNAi effects achieved in the gut would require a continuous input of high levels of dsRNA to persist. Production of adequate dsRNA in a transgenic plant and its delivery to the insect as food would resolve this technical problem [29].

Plant-mediated RNAi has been reported in lepidopteran and coleopteran plant pests [30], [31]. However, there is a more urgent need to develop this technique for application in insects for which no effective Bt (*Bacillus thuringiensis*) toxins are known. Insects in this category include phloem-sucking hemipteran pests, such as planthoppers, aphids and whitefly, which are highly destructive agricultural pests worldwide, causing millions of dollars' worth of yield loss and control costs [32].

The brown planthopper, *Nilaparvata lugens* Stål (Hemiptera: Delphacidae), is the most destructive insect pest of rice crops. The brown planthopper damages rice plants by directly sucking the phloem sap [33] and by acting as a vector for the transmission of the rice grassy stunt virus [34]. Although insecticide control of *N. lugens* has been a convenient option, indiscriminate usage has resulted in resistance, leading to a resurgence of the insect [35], besides creating serious environment pollution. Hence, genetic improvement of rice host resistance is a preferred alternative. Plant-mediated RNAi is a potential approach for controlling this insect pest of rice.

In this work, we cloned the *sid-1* and Argonaute genes and verified their expression in *N. lugens*. These genes could play important roles in the RNAi pathway in this insect. We also isolated genes encoding a hexose transporter (*NlHT1*), a carboxypeptidase (*Nlcar*) and a trypsin-like serine protease (*Nltry*), which are highly expressed in the midgut of *N. lugens*. dsRNA constructs targeting these genes were developed and transformed into rice plants. The results demonstrate that expression of the target genes was significantly suppressed in insects that fed on the transgenic plants.

## Results

### Cloning and characterization of the *sid-1* gene and the Argonaute gene in *N. lugens*

**Figure 1:**
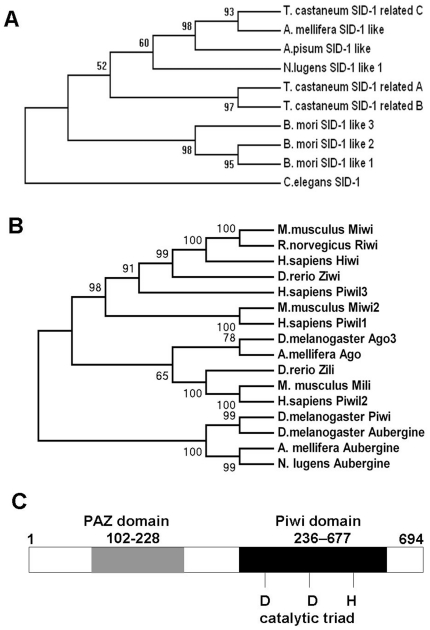
Phylogenetic relationships of the SID and Argonaute proteins from *N. lugens* and other species, and the structure of the *N. lugens* Aubergine protein. Phylogenetic analysis of (A) SID protein sequences, and (B) Argonaute protein sequences from *N. lugens* and other animals were conducted using MEGA 4.0; nodes with a bootstrap similarity of at least 50% are shown. (C) Conserved domains of Aubergine in *N. lugens*. It has the PAZ and Piwi domains that are typical motifs of Argonaute family proteins. The catalytic triad DDH is also shown. Sequence motifs were predicted by the NCBI Conserved Domains Server.

Because of the probable role of the SID protein in the uptake of dsRNA from the environment in *C. elegans*, we cloned the *sid-1* gene from *N. lugens*. The full-length cDNA of the *sid-1* gene is 2,119 bp long and contains an open reading frame (ORF) of 1,875 bp (GenBank accession no. JF915743), encoding a protein of 624 amino acids with a calculated molecular mass of 70.8 kDa and an isolectric point (pI) of 6.67 (Figure S1). Multiple alignment and phylogenetic analysis of the deduced amino acid sequences confirmed that this gene is a *sid-1* like gene, hence we named it *Nlsid-1* (*Nilaparvata lugens sid-1*). The phylogenetic tree of deduced amino acid sequences showed that the *N. lugens* SID-1 protein is most closely related to the *A. mellifera* SID-1 like protein (40% identity, Figure 1A).

To determine the potential for RNAi effects in *N. lugens*, we first obtained a sequence that is part of an Argonaute gene by random screening of a midgut cDNA library of *N. lugens*, then cloned the full-length cDNA corresponding to this Argonaute gene (Figure S2). The full-length cDNA of this *N. lugens* Argonaute gene is 3,446 bp long, including a 2,085 bp ORF (GenBank accession no. JF915742). The deduced protein was composed of 694 amino acid residues, with a molecular mass of 79.27 kDa and an isolectric point (pI) of 9.27. We compared 16 genes of the Argonaute family, including those previously reported from *Homo sapiens, Mus musculus, Rattus norvegicus, Danio rerio, D. melanogaster*, *Bombyx mori and A. mellifera*. As expected, the Argonaute gene in the brown planthopper belonged to an insect cluster and was closely related to Aubergine from *A. mellifera* (Figure 1B). Based on the phylogenetic analysis, we named the *N. lugens* gene *Nlaub* (*Nilaparvata lugens* Aubergine). Sequence motif analysis using the NCBI Conserved Domains Server identified the presence of typical PAZ and Piwi domains (Figure 1C), which are signature motifs of the Argonaute family proteins [36]. The predicted PAZ domain of *Nlaub* was 127 aa long, located at positions 102 to 228, while the Piwi domain of this protein was identified at residues 236–677. Degenerate catalytic motifs of the type Asp-Asp-Asp/His/Glu/Lys (known as DDH motifs), essential for slicer-mediated cleavage, were also identified in this protein.

### Analysis of the expression of *Nlsid-1* and *Nlaub* in *N. lugens*

**Figure 2:**
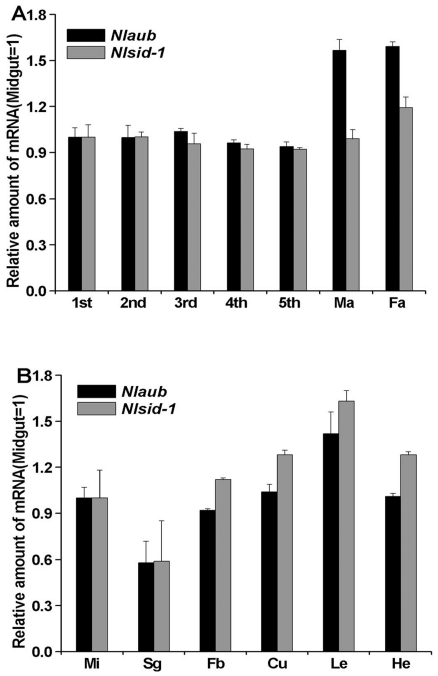
Expression of *Nlsid-1* and *Nlaub* in *N. lugens.* (A) Developmental expression of *Nlsid-1* and *Nlaub* in *N. lugens* from 1^st^ nymph to male adult (Ma) and female adult (Fa). (B) Tissue distribution of *Nlsid-1* and *Nlaub* in *N. lugens* 3^rd^ instar nymph. qRT-PCR analyses were performed using total RNA from midgut (Mi), salivary gland (Sg), fat body (Fb), cuticle (Cu), leg (Le), and head (He). Data shown are means ± standard errors (N = 3).

To probe the functions of the *Nlsid-1* and *Nlaub* genes, their mRNA levels were analyzed, using qRT-PCR, at various developmental stages of *N. lugens* insects, including nymphs from 1^st^ to 5^th^ instars, female adults and male adults. The developmental expression pattern revealed that *Nlsid-1* and *Nlaub* transcripts were present at all development stages. For both genes, mRNA levels were highest in female adults, but *Nlaub* mRNA had lower expression compared to that of *Nlsid-1* (Figure 2A). In order to further assess the tissue distribution of the transcripts, qRT-PCR was used to amplify *Nlsid-1* and *Nlaub* from cDNA of midgut, salivary gland, fat body, cuticle, leg, and head tissues. The results indicated that *Nlsid-1* and *Nlaub* were expressed in all six tissues tested (Figure 2B).

### Expression Profiles of *NlHT1*, *Nltry*, and *Nlcar*

Genes that are expressed in the *N. lugens* midgut were chosen as targets. Full length cDNA sequences of the transmembrane transporter gene *NlHT1* [37], the carboxypeptidase gene *Nlcar* and the trypsin-like serine protease gene *Nltry* were isolated from the *N. lugens* cDNA library. The sequences of *Nltry* and *Nlcar* are shown in Figure S3 and S4. The cDNA sequence of *Nltry (*GenBank accession no. JF915745) and the cDNA sequence of *Nlcar* (GenBank accession no. JF915744) have been deposited in GenBank.

**Figure 3:**
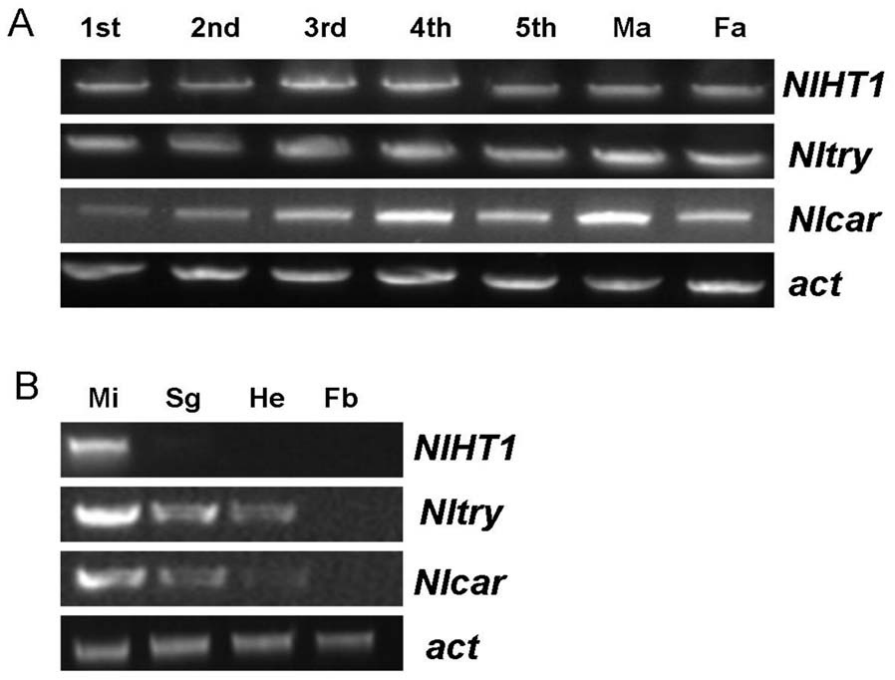
Transcription profiles of *NlHT1*, *Nltry*, and *Nlcar* at different developmental stages and in different tissues. (A) Transcript levels of the three genes in *N. lugens* developmental stages from 1^st^ nymph to male adult (Ma) and female adult (Fa). (B) Transcript levels in tissues of *N. lugens* 3^rd^ instar nymphs: midgut (Mi), salivary gland (Sg), fat body (Fb), and head (He).

As shown in Figure 3, each gene is transcribed in all developmental stages from 1^st^ to 5^th^ instars, female adults and male adults. The three genes show primary expression in midgut tissues with limited transcription in salivary glands, fat body and head.

### Development of dsRNA-transgenic rice plants

**Figure 4:**
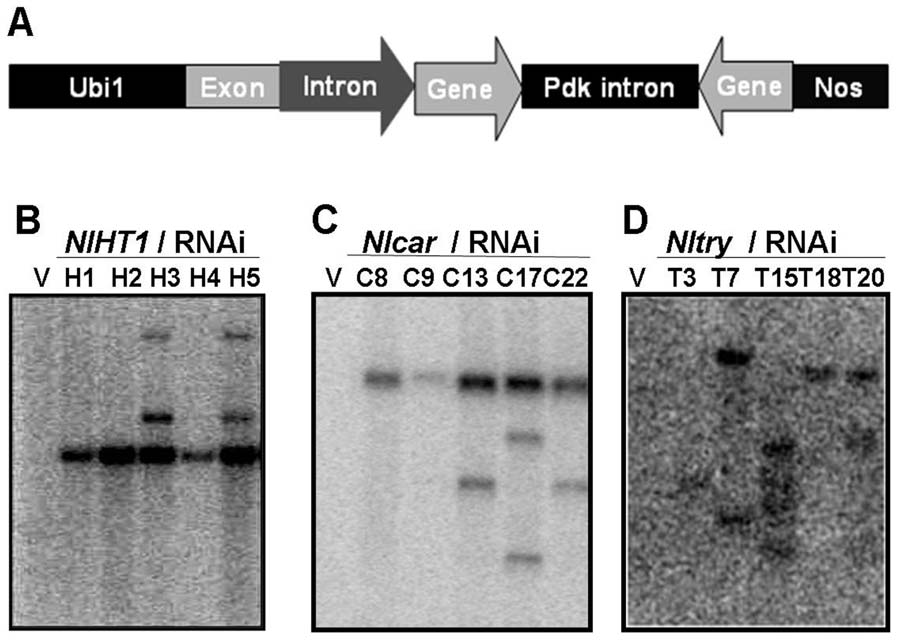
Structure of constructs for rice transformation and Southern blot analyses. (A) dsRNAi constructs (*NlHT1-*RNAi, *Nlcar*-RNAi and *Nltry*-RNAi) of target genes were developed under the control of the Ubi1 promoter and the nopaline synthase (Nos) terminator cassette. (B) Southern blot analysis of DraI-digested genomic DNA from leaves of *NlHT1*-RNAi-T0 transformants probed with part of the *NlHT1* coding sequence (lane V, DNA from the empty transformation vector plant; lanes 1–5, DNA from the H1, H2, H3, H4 and H5 transgenic lines). (C) Southern blot analysis of DraI-digested genomic DNA from leaves of *Nlcar*- RNAi-T0 transformants probed with part of the *Nlcar* coding sequence (lane V, DNA from the empty transformation vector plant; lanes 1–5, DNA from the C8, C9, C13, C17 and C22 transgenic lines). (D) Southern blot analysis of DraI-digested genomic DNA from leaves of *Nltry*- RNAi-T0 transformants probed with part of the *Nltry* coding sequence (lane V, DNA from the empty transformation vector plant; lanes 1–5, DNA from the T3, T7, T15, T18 and T20 transgenic lines).

**Figure 5:**
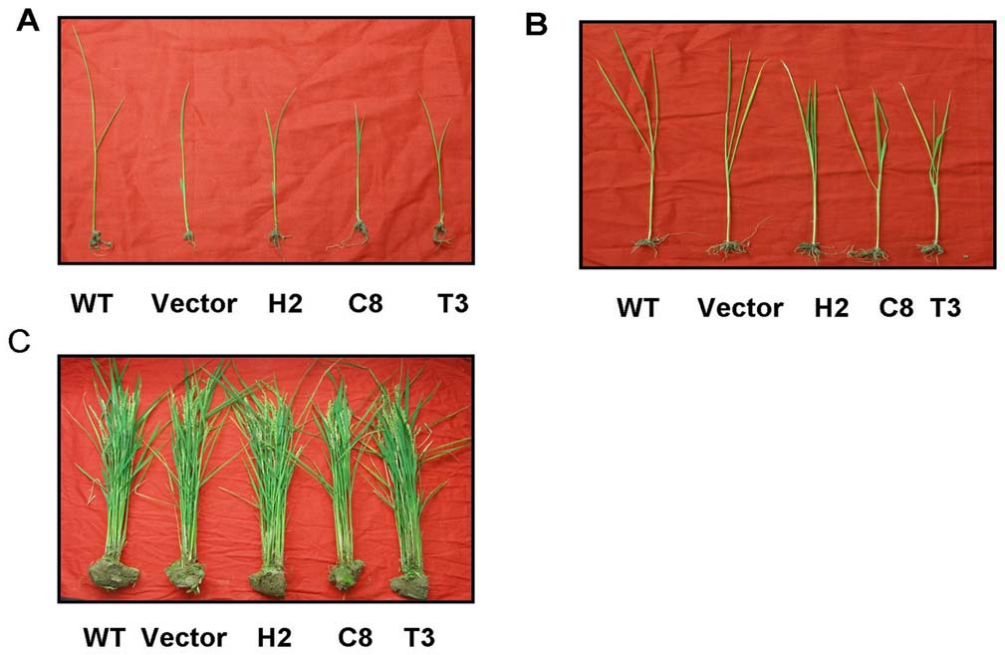
Growth phenotypes of wild type (WT) plants, empty transformation vector plants and transgenic lines. (A) Two-week-old seedlings. (B) Plants at the four leaf stage. (C) Mature plants.

Double strand RNA interference (dsRNAi) constructs *NlHT1*-RNAi, *Nlcar*-RNAi and *Nltry*-RNAi were developed for the three *N. lugens* genes *NlHT1*, *Nlcar* and *Nltry*, under the control of the Ubi1 promoter and the nopaline synthase (nos) terminator cassette [38], [39]. The genes were cloned at the BamHI site of the hygromycin gene expression cassette in the pCU vector of *Agrobacterium* (Figure 4A). Transgenic plants were generated by introducing the constructs into the japonica rice variety Hejiang 19 by *Agrobacterium tumefaciens*–mediated transformation, and genomic DNA was isolated from the hygromycin-tolerant transgenic rice plants. Positive transformants were detected by PCR of the hygromycin resistance gene, while wild type (WT) plants failed to show such amplification (data not shown). No significant morphological differences were found in these transgenic lines compared with WT plants (Figure 5). Southern blot analysis showed that the PCR-positive plants had 1–3 copies of the target coding sequences (Figure 4B–4D). Conversely, genomic DNA from transgenic plants carrying an empty vector failed to show any hybridization with the probes. Two independently transformed lines (H2 and H4, C8 and C9, T3 and T18) with a single-copy-insertion were chosen for *NlHT1*-RNAi, *Nlcar*-RNAi and *Nltry*-RNAi,respectively, for further analyses. Resistance and non-resistance to hygromycin were observed in progenies of these plants in the ratio 3∶1 (Table S2), confirming integration of the respective transgenes at a single locus in each case.

**Figure 6:**
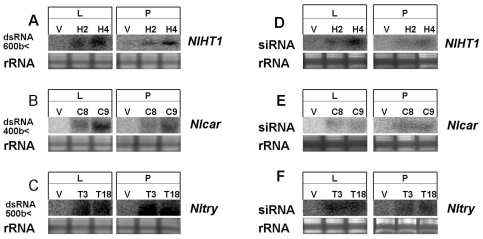
Northern blot analysis of the expression pattern of *NlHT1*, *Nlcar* and *Nltry* dsRNAs in different transgenic rice lines. RNA blots for the expression of (A) *NlHT1,* (B) *Nlcar* and (C) *Nltry* dsRNA in T1 lines homozygous for the dsRNA transgenes (H2 and H4, C8 and C9, T3 and T18 respectively) and in the absence of dsRNA in the T1 line homozygous for the empty transformation vector homozygous T1 line (V): L, leaf; P, phloem sap. (D) *NlHT1,* (E) *Nlcar* and (F) *Nltry* siRNAs were detected in the three dsRNA transgenic lines, respectively. Loading of equal amounts of RNA was confirmed by ethidium bromide staining.

RNA blot analysis showed that the *NlHT1*, *Nlcar* and *Nltry* dsRNAs were transcribed in the transgenic lines and some of them were processed to siRNAs (Figure 6). The expression and processing of dsRNAs for the target *N. lugens* genes in the transgenic rice lines provided dsRNA and siRNA molecules for potential ingestion by the insects.

### Suppression of target gene expression in *N. lugens* by ingestion of dsRNA- transgenic rice plants

**Figure 7:**
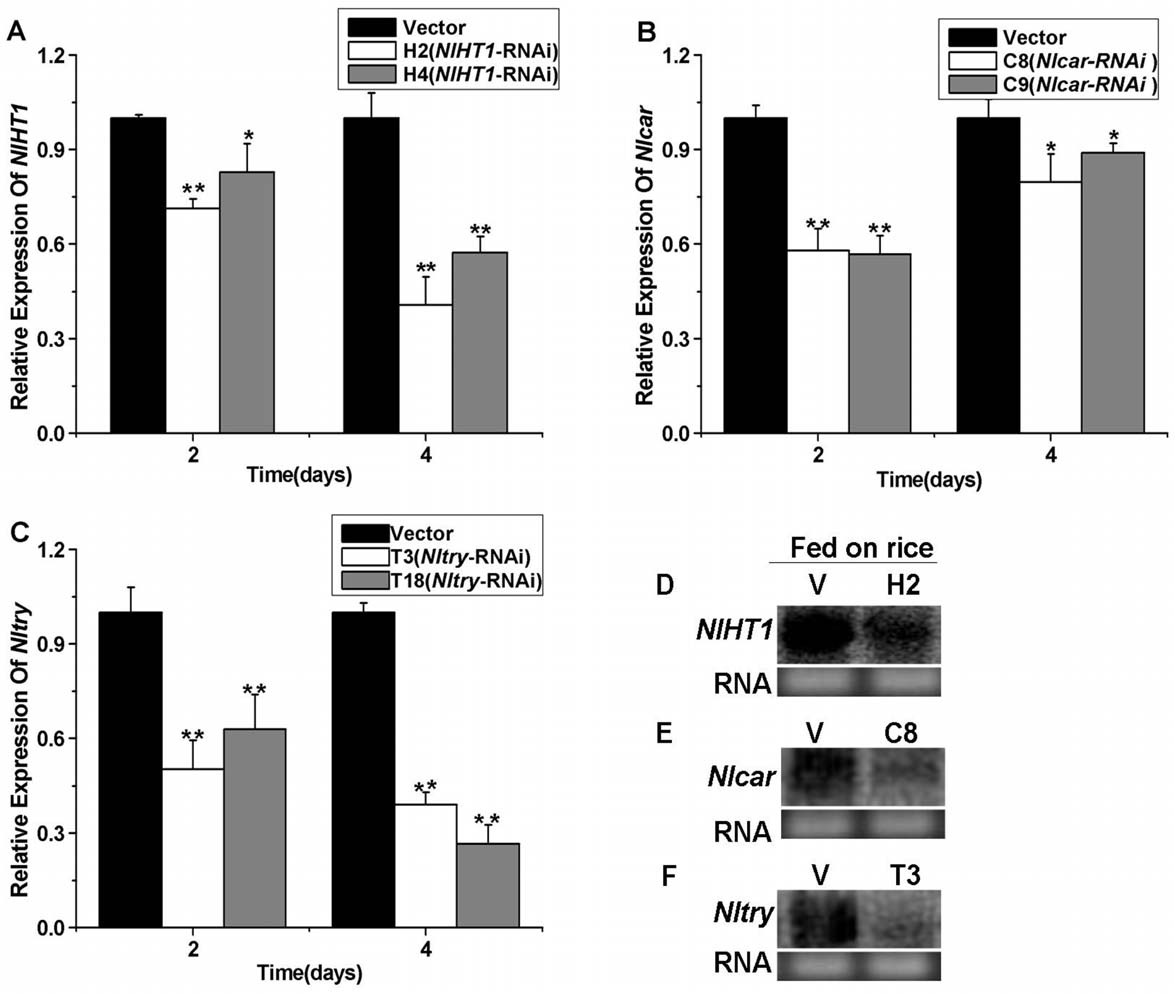
*NlHT1*, *Nlcar* and *Nltry* suppression in nymphs fed on transgenic plants. (A) *NlHT1* mRNA levels in the empty transformation vector and *NlHT1*-RNAi transgenic lines after feeding; nymphs were monitored by qRT-PCR after feeding on H2 and H4 phloem sap for 2 d and 4 d. (B) *Nlcar* mRNA levels in the empty transformation vector and *Nlcar*-RNAi transgenic lines after feeding; nymphs were monitored by qRT-PCR after feeding on C8 and C9 phloem sap for 2 d and 4 d. (C) *Nltry* mRNA levels in the empty transformation vector and *Nltry*-RNAi transgenic lines after feeding; nymphs were monitored by qRT-PCR after feeding on T3 and T18 phloem sap for 2 d and 4 d. Statistical analysis of mRNA levels was performed with student t-test.(*, P<0.05; **, P <0.01.) (D) Northern blot of *NlHT1* transcripts of 3^rd^ instar nymphs fed on the empty transformation vector and H2 transgenic plants for 4 d. (E) Northern blot of *Nlcar* transcripts of 3^rd^ instar nymphs fed on the empty transformation vector and C8 transgenic line for 4 d. (F) Northern blot of *Nltry* transcripts of 3^rd^ instar nymphs fed on the empty transformation vector and T3 transgenic line for 4 d.

Third instar nymphs, previously reared on susceptible rice (cultivar Taichung Native 1, TN1) plants, were transferred onto the dsRNA-transgenic plants and allowed to feed for 2 to 4 days. The transcript levels of the target genes in *N. lugens* were then detected using qRT-PCR. In nymphs fed on the H2 and H4 lines, *NlHT1* transcript levels began to decrease on day 2 and were reduced by 59.3% in H2 and 42.7% in H4 on day 4 (Figure 7A). A similar experiment was performed on the *Nlcar* and *Nltry* dsRNA-transgenic plants. The qRT-PCR results revealed that the mRNA transcripts of *Nlcar* and *Nltry* after ingestion of the respective dsRNAs were reduced on days 2 and 4. The maximum reductions in the *Nlcar* transcript level, of 42.1% for the C8 line and 43.3% for the C9 line, occurred on day 2 (Figure 7B). The maximum reductions in the *Nltry* transcript levels, of 61% for the T3 line and 73.3% for the T18 line, occurred on day 4 (Figure 7C). The northern blotting results confirm that the brown planthopper nymphs fed on H2 plants had less *NlHT1* transcript compared to nymphs on the plants transformed with an empty transformation vector (Figure 7D). Similarly, the nymphs from fed on C8 and T3 plants had lower *Nlcar* and *Nltry* expression levels than those on the plants transformed with an empty transformation vector (Figure 7E–7F). Figure 7A–7C also shows that the RNAi efficiency for *Nltry* was significantly higher (p<0.01, t-test) than for *NlHT1* and *Nlcar* on day 4. The results show that ingestion of dsRNA-transgenic rice plants expressing dsRNA and siRNA is an effective way to trigger RNA interference in the Hemipteran insect *Nilaparvata lugens*.

We also assessed the performance of BPH on the transgenic plants(*NlHT1*-RNAi, *Nlcar*-RNAi and *Nltry*-RNAi and an empty transformation vector). The significant lethal phenotype was not observed (Figure S5).

## Discussion

RNAi occurs widely in eukaryotic organisms and previous studies have demonstrated that feeding-based RNAi can specifically induce an RNAi response in several insect species, such as *S. exigua, H. armigera* and *D. virgifera virgifera*. In Lepidoptera, trends that are found are that RNAi is particularly successful in the family Saturniidae [12]. In this study, we showed that the target genes in the midgut of the hemipteran *N. lugens* can be suppressed by dsRNA transgenic plant-mediated RNA interference.

### Identification of SID-1 and Argonaute genes in *N. lugens*

SID-1 is required for the uptake and spread of gene-silencing signals between tissues, thereby mediating a systemic RNAi effect in *C. elegans* [40], [41]. In this study, we have identified a *sid-1* gene (*Nlsid-1*) in *N. lugens*. *Sid-1-*like genes have been found in many insects [3], and expression of these genes has been confirmed in *Tribolium castaneum*, *Apis mellifera* and *Schistocerca americana* [7], [42], [43]. In Hemiptera, a *sid-1-*like gene of the cotton aphid (*Aphis gossypii*) has been cloned and the topological structure of the SID-1 protein suggested a possible role in dsRNA uptake [20]. To determine whether SID-1 exists in *N. lugens,* we cloned a cDNA sequence of the *sid-1* gene from the brown planthopper and qRT-PCR results showed that *Nlsid-1* was expressed in various tissues of nymphs and adult insects. The deduced protein showed the closest relationship with the honey bee (*Apis mellifera*) SID-1 protein (40% identity). The honey bee SID-1, a putative transmembrane protein encoded by *AmSid-1*, is necessary for the uptake of systemically administered dsRNA and subsequent gene silencing [42]. Consistent with conservation and expression of the dsRNA channel protein gene *sid-1* in grasshopper, systemic application of dsRNA leads to specific, long-term and phenotypically visible reduction of endogenous grasshopper v gene activity [43].*Nlsid-1* may therefore perform an essential role in the uptake of dsRNA in the midgut of *N. lugens* and in any RNAi effect which is subsequently induced. Further research should establish whether there is a direct correlation between increased *Nlsid-1* expression and the silencing effect in *N. lugens*.

Members of the Argonaute protein family have demonstrated activity in both transcriptional and post-transcriptional gene silencing. Ago proteins play important roles in RNAi because they can bind siRNAs as well as miRNAs and are involved in the suppression of specific target RNAs by either mRNA breakdown or inhibition of translation [44], [45]. In insects, the Ago and Piwi subfamilies both belong to the Argonaute protein family. Phylogenetic analysis indicated that the Piwi subfamily could be divided into three clusters, Piwi, Aubergine (Aub), and Ago3. Aubergine has been demonstrated to be required in RISC assembly in vitro [46] and in RNAi in oocytes in vivo [47]. In *Drosophila melanogaster*, the overexpression of AUB protein interferes with some crucial component or function of the RNAi pathway in somatic tissues [48]. Interestingly, AUB was thought to have a major role in silencing repetitive elements at the germ-tissue level [49]. In this study, we isolated an *N. lugens* Piwi subfamily gene *Nlaub*, which shares high identity (50%) with the *A. mellifera* Aubergine gene (GenBank accession no. ACV84377). The deduced amino acid sequence of *Nlaub* demonstrated the presence of the PAZ and Piwi domains. The Piwi domain is essential for slicing events in RNAi [36]. The presence of *Nlaub* gene transcripts in different developmental stages and tissues of *N. lugens* suggests a possible role in dsRNA-processing for the RNAi pathway in this insect.

### Ingestion of dsRNA-transgenic rice plants induces target gene repression in *N. lugens* nymphs

Most experiments on RNAi in insects reported to date have involved injecting dsRNA directly into the body, although development of a robust dsRNA feeding technique is a prerequisite for application of RNAi to crop protection against insect pests. However, there have been some exceptions, including the following examples: Turner et al. [11] demonstrated RNAi effects after oral delivery of dsRNA to *Epiphyas postvittana* to silence a gut carboxylesterase gene (*EposCXE1*); only a local RNAi effect was required for repression. Zhou et al. found that knockdown of a gene encoding a caste-regulatory hexamerin storage protein could be achieved through voluntary feeding of high-dose dsRNA to *Reticulitermes flavipes* [13]. In contrast to these positive results, in an earlier study attempts to feed dsRNA to the lepidopteran *Spodoptera litura* to knockdown the midgut aminopeptidase-N gene in larvae did not generate an RNAi response [50]. Application of RNAi technology through transgenic plants to deliver dsRNA to herbivorous insects has only recently been realized in two chewing insects, cotton bollworm (*Helicoverpa armigera*; Lepidoptera) and western corn rootworm (WCR; *Diabrotica virgifera virgifera*; Coleoptera) [30], [31].

*N. lugens* feeds on plants by sucking phloem sap through its stylet mouthparts and causes substantial physiological damage to rice [51]. Sugars and amino acids are present in rice phloem sap at high concentrations. Some of the nutrients are absorbed into the planthopper body through the midgut cells, and the rest are excreted as honeydew [52]. Price et al. [37] reported that *NlHT1*, which is expressed at highest levels in the midgut, plays an important role in glucose transport from the gut, and in carbon nutrition *in vivo*. Digestion by serine proteases also plays an important role in *N. lugens* nutrition [53], since proteins are major components of plant phloem sap too; for example, cucurbit exudate contains high concentrations of proteins, up to 100 mg ml^−1^ [54]. The carboxypeptidase gene *Nlcar*, the trypsin-like serine protease gene *Nltry* and the *NlHT1* gene are primarily expressed in the midgut of *N. lugens* (Figure 3). In this study, these three genes were selected for the development of dsRNA constructs which were transferred into rice plants. In the transgenic plants, some long dsRNAs were processed into siRNAs and both dsRNA and siRNA were present in the phloem sap (Figure 6). Ingestion of transgenic rice plants producing *NlHT1*, *Nlcar* and *Nltry* dsRNA was shown to suppress the expression of the three genes in *N. lugens*. Reduction of mRNA expression by 40% to 70% was observed (Figure 7), demonstrating the effectiveness of delivering dsRNA through the feeding of transgenic plants for the brown planthopper. Two research groups have demonstrated that artificial diet-feeding [55] and direct injection [56] of dsRNA can specifically induce an RNAi response in *N. lugens.* By injecting dsRNAs into *N. lugens*, Liu et al. [56] obtained a decrease of 25% to 40% of the expression of *calreticulin*, *cathepsin-B* and *Nlß2* genes. The results reported in the present paper show that, in addition to injection and artificial diet-feeding, ingestion of dsRNA in host plants can induce inhibition of target gene expression in *N. lugens*.

Interestingly, although the transcription levels of the target genes were suppressed (Figure 7), we did not observe a significant lethal phenotype (Figure S5). In root knot nematode (RKN; *Meloidogyne* spp.), a splicing factor and integrase have been successfully down-regulated by transgenic plants expressing dsRNAs, resulting in almost complete resistance [57]. In another study, transgenic *Arabidopsis* expressing dsRNAs specific to genes encoding a nematode secretory peptide (16D10) that stimulates root growth successfully knocked down expression of genes in four major root knot nematodes, resulting in effective levels of resistance against the RKN species [58]. A further study has shown the feasibility of suppressing a root knot nematode transcription factor, *MjTis11*, *in vivo* through plant-expressed dsRNA. Those transgenic plants that induced the silencing of *MjTis11*, however, did not result in a lethal phenotype [59]. For *N. lugens* in our study, one possible reason for such a result is that the amount of dsRNA-uptake by the insects was not sufficient. Alternatively, the experimental system was not sufficient for maximum penetrance of the RNAi effects to cause a lethal phenotype. Another possible explanation is that the genes of interest may belong to multi-gene families in which other members may compensate for the suppression of one member [52], [60]. It is also possible that the target genes are not physiologically significant enough to induce mortality when silenced. The susceptibility of different target genes to RNAi in *N. lugens* shows considerable variation and further investigation is required. Although possible systems for dsRNA uptake and RNAi are present and target genes can be knocked down, many factors must be optimized in order to develop an RNAi approach for countering insect pest species such as *N. lugens*. This study has demonstrated the presence of a system for dsRNA uptake and the potential for knockdown of target gene expression by RNAi, but other factors to consider include the dsRNA fragment length, the relative susceptibilities of different insect developmental stages, and the nucleotide sequences selected as targets. It has been proved in *C. elegans* that RNAi effects are species-specific because knockdown experiments and identification of lethal phenotypes has not led to a universal set of ‘nematode target genes’ that are helpful for protection against plant parasitic nematodes [29]. Therefore, in order for the transgenic plant approach to be successful, appropriate target genes must be selected individually for each insect pest species. In addition, the specificity of ideal genes for inhibition in transgenic plants is an important consideration for the use of this technology in practical applications, since effects on non-target insects should be minimized.

## Materials and Methods

### Plant and Insect Material

The rice brown planthopper (BPH; *Nilaparvata lugens*) was obtained from rice fields in Zhejiang Province, China, and reared on two to three month old rice plants of the susceptible variety TN1, under controlled environmental conditions (70–80% r.h., 25±2°C, 16 h light/8 h dark). Four week old plants were used for the feeding experiments. For each feeding experiment, synchronous nymphs were selected and divided into groups, each group containing 30–40 individuals. After feeding on transgenic plants for the number of days indicated in the figures, midguts of nymphs were taken for further analysis.

### Cloning of *N. lugens* genes

A cDNA library of *N. lugens* was constructed with RNA isolated from dissected midguts using the Creator SMART cDNA Library Construction Kit (CLONTECH) according to the manufacturer's recommendations. The cDNA was size-fractionated to remove fragments of <500 bp, directionally ligated into Lambda TriplEx2 vectors (BD-Clontech) and packaged into lambda phages (Stratagene). Individual clones were randomly selected and sequenced over 5′ and 3′ ends. Sequences were annotated by BLAST similarity searches against the GenBank database. Genes with similarity to the carboxypeptidase gene *Nlcar*, the trypsin-like serine protease gene *Nltry* and the Argonaute gene *Nlaub* were identified and the entire coding sequence was reamplified by PCR from *N. lugens* midgut-specific first-strand cDNA. For the *sid-1* like gene, a TBLASTN search of the BPH expressed sequence tags (ESTs) database, using the *Acyrthosiphon pisum* similar to SID1-like protein [GenBank: XP_001951907] as query, revealed one SID1-like EST [GenBank: S_NLNA6932].

To obtain the full-length sequence of each truncated sequence from *N. lugens*, 5′ and 3′ RACE amplifications were performed using a 5′-Full RACE Kit and 3′-Full RACE Core Set Ver. 2.0 (TaKaRa) following the manufacturer's instructions. For 5′-RACE, gene-specific primers were designed based on sequencing, and an external reverse and a nested primer (Table S1) were used. For 3′-RACE, the cDNA was then amplified by nested PCR with the external forward primer and nested forward primer (Table S1) with ExTaq DNA polymerase (TaKaRa). The first round PCR cycling conditions were: 94°C for 3 min, followed by 20 cycles of 94°C for 30 sec, annealing at 55°C for 30 sec, and 72°C for 2 min, then an additional final elongation step of 72°C for 10 min. Nested PCR amplification was performed under the same conditions for 25 cycles. Purified PCR products were ligated to pGEM®-T Easy Vector Systems (Promega) and 3 to 5 positive colonies were sequenced.

### Vector construction and plant transformation

For the RNA interference (RNAi) vectors of *NlHT1, Nlcar* and *Nltry*, DNA fragments with different restriction enzyme sites at both ends were amplified by PCR using RNAi1 and RNAi2 primer pairs (Table S1). The two PCR fragments were inserted at inverted repeats into the pKANNIBAL vector to generate a hairpin RNAi construct, which was then cloned into the binary vector pCU BamHI site [61]. The final RNAi vector was introduced into *Agrobacterium tumefaciens* (strain EHA105) by electroporation. Transgenic rice plants were generated by *Agrobacterium*-mediated transformation of rice calli, as previously described [62]. Transformed calli were selected by hygromycin resistance, and the transgenic plants were regenerated from the transformed calli. Regenerated transgenic rice plants were grown in a greenhouse.

### Southern blot analysis

Genomic DNA was isolated from hygromycin-tolerant and empty transformation vector plants using the method of Chakraborti et al. [63]. The DNA from the empty transformation vector plants was used as a negative control. Fifteen microgram portions of the genomic DNA were digested with DraI, and the digested DNA was subjected to electrophoresis on a 0.8% agarose gel, then transferred to a Hybond N^+^ membrane (Amersham). The probe was labeled with [α-^32^P]dCTP using the Prime-a-Gene labeling system (Promega), and the membranes were hybridized for at least 10 h at 65°C with the labeled probe. Following incubation, the blot was washed with 2× SSC/0.5% SDS for 15 min at 65°C followed by 1× SSC/0.1% SDS for 15 min at 65°C.

### Northern blot analysis

Total RNAs were isolated from *N. lugens* or plant tissues by Trizol reagent (Invitrogen).About 20 µg of total RNA was separated on a denaturing 1.5% formaldehyde agarose gel. Loading of equal amounts of RNA was confirmed by ethidium bromide staining. The formaldehyde gels were blotted onto a Hybond N^+^ membrane (Amersham) and hybridized with [α-^32^P] dCTP labeled *N. lugens* genes probe. The membranes were washed with 2× SSC/0.1% SDS for 15 min at 65°C followed by 1× SSC/0.1% SDS for 15 min at 65°C.

### Small RNA analysis

siRNA detection was performed as previously described [64]. Electrophoresis was performed in 3% agarose gels in the presence of formaldehyde. Blots of siRNAs were prehybridized at 58°C for 2 h, and hybridizations were performed at 42°C for 16 h. The blots were washed with 2× SSC/0.5% SDS for 3×10min at 37°C followed by 2× SSC/0.2% SDS for 10 min at 42°C. The primers (18 and 21 nucleotides) were used as size standard and hybridization controls.

### Quantitative real-time PCR analysis

Total RNA was isolated from dissected tissues by Trizol reagent (Invitrogen). First-strand cDNA was synthesized at 42°C from total RNA using M-MLV reverse transcriptase (Fermentas). Expression of selected *N. lugens* genes and ß-Actin (EU179846) [65] was quantified by quantitative RT-PCR, using an RG-6000 rotary analyzer (Corbett Research) and appropriate primers (Table S1) together with 1 µg of RNA per reaction. The following pair of primers was used for ß-Actin (which served as an internal control to normalize differences in recovery between samples: 5′-TGGACTTCGAGCAGGAAATGG-3′ and 5′- ACGTCGCACTTCATGATCGAG-3′).

### Phylogenetic Analysis

The deduced amino acid sequences of SID-1 and Argonaute were aligned using ClustalW. Accession numbers of SID-1s used for sequence alignment and phylogenetic analyses were as follows: *N. lugens* SID-1 like, JF915743; *B. mori* SID-1 like 1, BAF95805; *B. mori* SID-1 like 2, BAF95807; *B. mori* SID-1 like 3, BAF95806; *T. castaneum* SID-1 related A, NP_001099012; *T. castaneum* SID-1 related B, NP_001103253; *T. castaneum* SID-1 related C, NP_001099128; *A. mellifera* SID-1 like, XP_395167; *A. pisum* SID-1 like, XP_001951907; and *C. elegans* SID-1, AAL78657. Accession numbers of Argonaute used for sequence alignment and phylogenetic analyses are as follows: *N. lugens* Aubergine, JF915742; *M. musculus* Mili, BAA93706; *M. musculus* Miwi, BAA93705; *M. musculus* Miwi2, AAN75583; *D. rerio* Ziwi, NP_899181; *D. rerio* Zili, ACH96370; *R. norvegicu*s Riwi, NP_001102323; *H. sapiens* Hiwi, AAC97371; *H. sapiens* Piwil1, BAC81341; *Homo sapiens* Piwil3, BAC81343; *H. sapiens* Piwil2, BAC81342; *D. melanogaster* Piwi, AAD08705; *D. melanogaster* Ago3, ABO26294; *D. melanogaster* Aubergine, NP_476734; *A. mellifera* Aubergine, ACV84377; *A. mellifera* Argonaute, ACV84372. Phylogenetic analyses were conducted by the neighbor-joining method using MEGA 4.0. Bootstrap values were assessed with 1000 replicates.

### BPH survival rate determination

For the plant feeding experiment, synchronous nymphs were selected and divided into groups. Pots, each containing one plant, were individually covered with plastic cages (diameter 12 cm, height 35 cm) into which 20–30 individuals were released. After feeding on plant diets for the indicated number of days, midguts of nymphs were taken for further analysis. Tests of statistical significance were performed with Student's t-test in Excel.

The survival rates of BPH nymphs on the transgenic plants(transformed with *NlHT1*-RNAi, *Nlcar*-RNAi and *Nltry*-RNAi constructs and with the empty transformation vector)were determined. Pots, each containing one plant, were individually covered with plastic cages (diameter 9 cm, height 12 cm) into each of which 10 newly hatched BPH nymphs were released. The number of surviving BPH nymphs on each plant was recorded every day until 12 days after the introduction of the herbivore. The experiment was repeated 10 times.

## Supporting Information

Figure S1 ***Nlsid-1*** **nucleotide and deduced amino acid sequences. Transmembrane regions, as predicted by TMHMM, are underlined (TM 1–7).**
(TIF)

Figure S2 ***Nlaub*** **nucleotide and deduced amino acid sequences.** The deduced amino acid sequence is shown below the cDNA sequence. The PAZ and Piwi domains are underlined.
(TIF)

Figure S3 **The nucleotide and deduced amino acid sequences of the** ***N. lugens*** **trypsin-like serine protease gene (*Nltry*).**
(TIF)

Figure S4 **The nucleotide and deduced amino acid sequences of the** ***N. lugens*** **carboxypeptidase gene (*Nlcar*).**
(TIF)

Figure S5 **Mean survival rates of BPH nymphs fed on transgenic plants transformed with empty transformation vector (Vector),** ***NlHT1*** **-RNAi (H2),** ***Nlcar*** **-RNAi (C8), and** ***Nltry*** **-RNAi (T3).**
(TIF)

Table S1 **Primer sequences used in the present study.**
(DOC)

Table S2 **Phenotypic segregation analysis in progeny (T_1_ generation) of six RNAi transgenic lines.**
(DOC)
