# Supplementary figures and images for: Knockdown of Midgut Genes by dsRNA-Transgenic Plant-Mediated RNA Interference in the Hemipteran Insect Nilaparvata lugens
Source: PLoS One. 2011 May 31;6(5):e20504. doi: 10.1371/journal.pone.0020504 (PMC3105074; doi:10.1371/journal.pone.0020504)

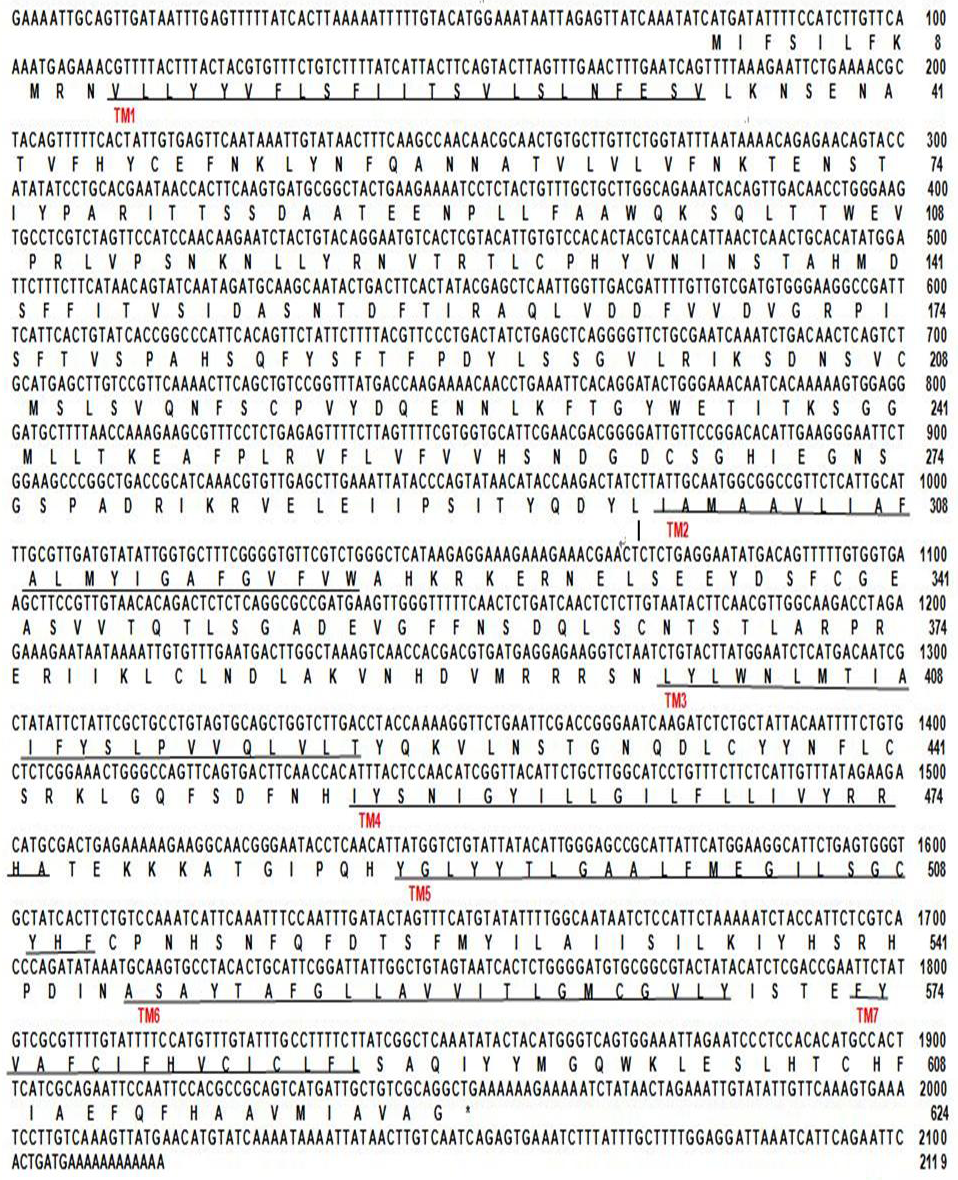

Supplement: Figure S1 — Nlsid-1 nucleotide and deduced amino acid sequences. Transmembrane regions, as predicted by TMHMM, are underlined (TM 1–7). (TIF) [file pone.0020504.s001.tif]

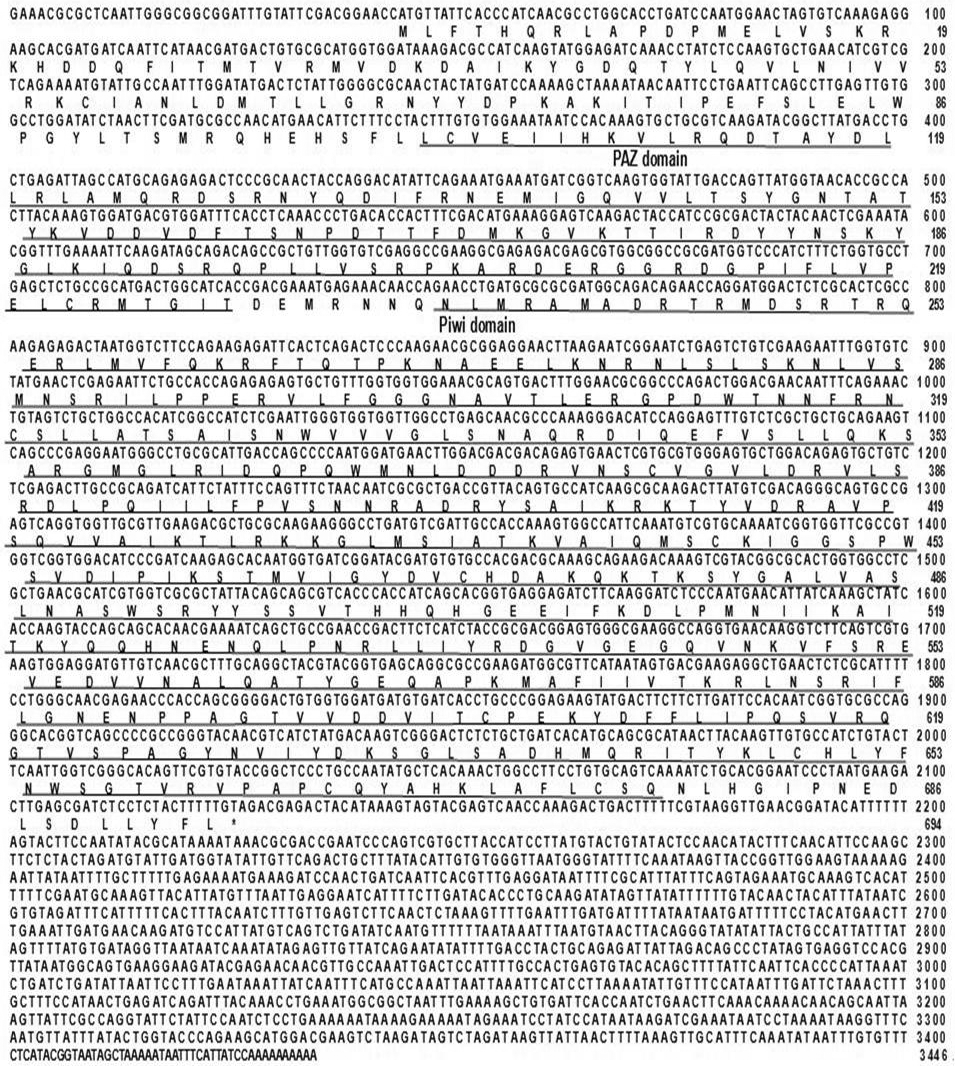

Supplement: Figure S2 — Nlaub nucleotide and deduced amino acid sequences. The deduced amino acid sequence is shown below the cDNA sequence. The PAZ and Piwi domains are underlined. (TIF) [file pone.0020504.s002.tif]

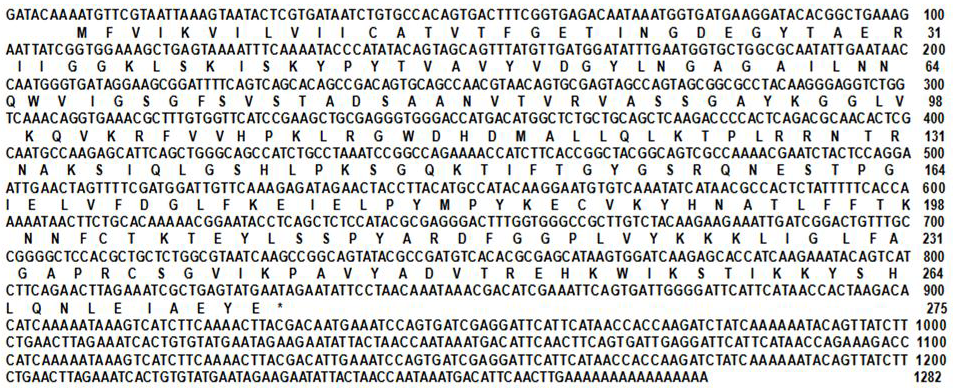

Supplement: Figure S3 — The nucleotide and deduced amino acid sequences of the N. lugens trypsin-like serine protease gene (Nltry). (TIF) [file pone.0020504.s003.tif]

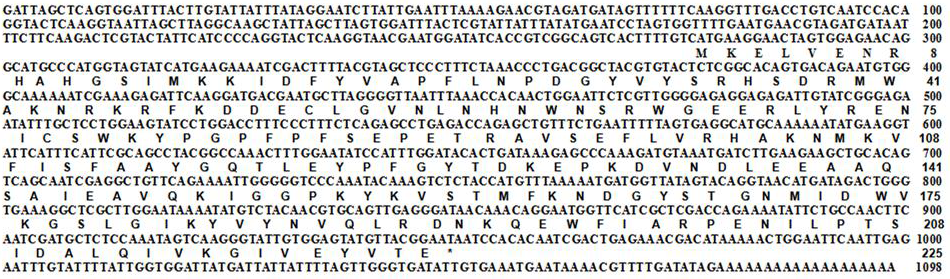

Supplement: Figure S4 — The nucleotide and deduced amino acid sequences of the N. lugens carboxypeptidase gene (Nlcar). (TIF) [file pone.0020504.s004.tif]

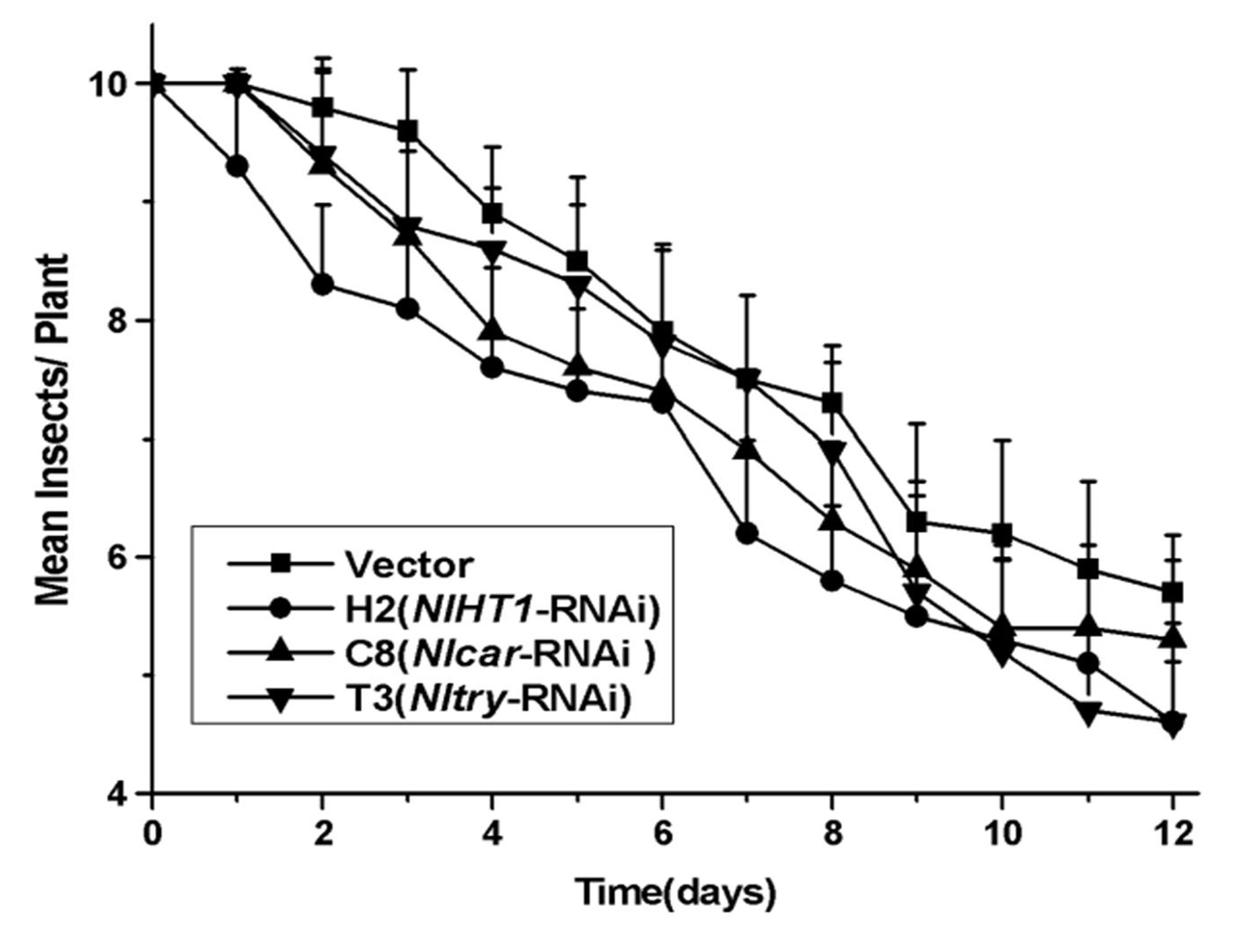

Supplement: Figure S5 — Mean survival rates of BPH nymphs fed on transgenic plants transformed with empty transformation vector (Vector), NlHT1 -RNAi (H2), Nlcar -RNAi (C8), and Nltry -RNAi (T3). (TIF) [file pone.0020504.s005.tif]
