# Supplementary material for: Knockdown of Midgut Genes by dsRNA-Transgenic Plant-Mediated RNA Interference in the Hemipteran Insect Nilaparvata lugens
Source: PLoS One. 2011 May 31;6(5):e20504. doi: 10.1371/journal.pone.0020504 (PMC3105074; doi:10.1371/journal.pone.0020504)
Supplement: Table S1 — Primer sequences used in the present study. (DOC) [file pone.0020504.s006.doc]

**Table S1. Primer sequences used in the present study**.

| Gene name | Primer name | Sequence 5’-3’ |
| --- | --- | --- |
| RNAi vector construction | | |
| *NlHT1* | RNAi1- forward | CCTCGAGGGATCCTGCCTATGAGTTGGATGCTG |
|  | RNAi1- reverse | CGAATTCGTGTCATTTGAGGGGTTGCT |
|  | RNAi2- forward | CTCTAGAGGATCCTGCCTATGAGTTGGATGCTG |
|  | RNAi2- reverse | CATCGATGTGTCATTTGAGGGGTTGCT |
| *Nlcar* | RNAi1- forward | CCTCGAGGGATCCATGAAGGAACTAGTGGAGAA |
|  | RNAi1- reverse | CGGTACCTTATTCCGTAACATACTCCA |
|  | RNAi2- forward | CTCTAGAGGATCCATGAAGGAACTAGTGGAGAA |
|  | RNAi2- reverse | CAAGCTTTTATTCCGTAACATACTCCA |
| *Nltry* | RNAi1- forward | CCTCGAGGGATCCATGTTCGTAATTAAAGTAAT |
|  | RNAi1- reverse | CGAATTCCTATTCATACTCAGCGATTT |
|  | RNAi2- forward | CTCTAGAGGATCCATGTTCGTAATTAAAGTAAT |
|  | RNAi2- reverse | CAAGCTTCTATTCATACTCAGCGATTT |
| Real-time PCR | | |
| *NlHT1* | Forward | Ggatcagatggaagcagagc |
|  | Reverse | ttgcagacttggactcatcg |
| *Nlcar* | Forward | TCCTGGACCTTTCCCTTTCT |
|  | Reverse | AACAGCCTCGATTGCTGACT |
| *Nltry* | Forward | GTTGATTCGTTTTGGCGACT |
|  | Reverse | GGTGGTCTGGTCAAACAGGT |
| RACE |  |  |
| *Nlaub* | 5’race_gsp | ATAAGGATGGTAAGCACGACTGGG |
|  | 5’race_ngsp | TCGCTCAAGTCTTCATTAGGGA |
|  | 3’race_gsp | CTCCAACATACTTTCAACATTC |
|  | 3’race_ngsp | TCCCTAATGAAGACTTGAGCGA |
| *Nlsid-1* | 5’race_gsp | CGCCTGAGAGAGTCTGAGTT |
|  | 5’race_ngsp | CCTCTTATGAGCCCAGACGAACACCC |
|  | 3’race_gsp | GTTGTAACTCAGACTCTCTCAGGC |
|  | 3’race_ngsp | GCGACTGAGAAAAAGAAGGCAACG |
