# Supplementary material for: Knockdown of Midgut Genes by dsRNA-Transgenic Plant-Mediated RNA Interference in the Hemipteran Insect Nilaparvata lugens
Source: PLoS One. 2011 May 31;6(5):e20504. doi: 10.1371/journal.pone.0020504 (PMC3105074; doi:10.1371/journal.pone.0020504)
Supplement: Table S2 — Phenotypic segregation analysis in progeny (T1 generation) of six RNAi transgenic lines. (DOC) [file pone.0020504.s007.doc]

**Table S2.** Phenotypic segregation analysis in progeny (T_1_ generation) of six RNAi transgenic lines.

| Line | Copy number of transgene | Number of seeds germinated | hyg^PCR+^ | hyg^PCR-^ | χ^2^ (3:1)^a^ |
| --- | --- | --- | --- | --- | --- |
| blank vector | 1 | 30 | 22 | 8 | 0.044^a^ |
| H2 | 1 | 35 | 24 | 11 | 0.771^a^ |
| H4 | 1 | 23 | 20 | 3 | 1.753^a^ |
| C8 | 1 | 6 | 4 | 2 | 0.222^a^ |
| C9 | 1 | 37 | 29 | 8 | 0.225^a^ |
| T3 | 1 | 15 | 13 | 2 | 1.089^a^ |
| T18 | 1 | 24 | 19 | 5 | 0.222^a^ |

^a^The difference from the expected 3:1 segregation is not signiﬁcant at P <0.05.
